# The significance of transrectal ultrasound and urologist_dually guided pelvic floor muscle exercise in improving urinary continence after radical prostatectomy

**DOI:** 10.1186/s40001-023-01133-3

**Published:** 2023-05-13

**Authors:** Yin Huaqi, Du Zheng, Ma Yongkang, Zhao Shiming, Sun Zhenghui, Wang Zhiwei, Li Congyu, Li Qian, Dong Bingqi, Zhu Mingkai, Zhu Chaoshuai, Peng Jiangshan, Yang Tiejun

**Affiliations:** 1 Department of Urology, The Affiliated Cancer Hospital of Zhengzhou University, Henan Cancer Hospital, No.127, Dong Ming Road, Zhengzhou, 450000 China; 2 Department of Ultrasound, The Affiliated Cancer Hospital of Zhengzhou University, Henan Cancer Hospital, No.127, Dong Ming Road, Zhengzhou, 450000 China

**Keywords:** Prostate cancer, Urinary continence, Transrectal ultrasound, PFME, Radical prostatectomy

## Abstract

**Background:**

To determine whether transrectal ultrasound and urologist_dually guided pelvic floor muscle exercise is associated with immediate, early and long-term urinary continence after radical prostatectomy.

**Materials and methods:**

Data from 114 patients with localized prostate cancer (PC) who underwent RP at Henan Cancer Hospital from November 2018 to April 2021 were included in the retrospective study. Of the 114 patients, 50 patients in the observation group underwent transrectal ultrasound and urologist_dually guided PFME, and 64 patients in the control group underwent verbally_guided PFME. Contractile function of the external urinary sphincter was in the observation group was evaluated. The immediate, early and long-term urinary continence rates were assessed in both groups, and the factors affecting urinary continence were analyzed.

**Results:**

The urinary continence rate at 2 weeks and 1, 3, 6 and 12 months in the observation group after RP was significantly higher than that in the control group (52.0% vs. 29.7%, 70.0% vs. 39.1%, 82% vs. 57.8, 88% vs. 70.3%, 98.0 vs. 84.4%, *p* < 0.05). The contractile function of the external urinary sphincter was obviously correlated with urinary continence at multiple visits after RP, except for the 12-month visit. Transrectal ultrasound and urologist-dually guided PFME was verified to be an independent positive factor for urinary continence at 2 weeks and 1, 3, 6 and 12 months using logistic regression analysis. However, TURP was a negative factor for postoperative urinary continence at different times.

**Conclusions:**

Transrectal ultrasound and urologist_dually guided PFME had a significant role in improving immediate, early and long-term urinary continence after RP and acted as an independent prognostic factor.

## Introduction

Prostate cancer (PC), one of the most common cancers in men, exhibits an increasing incidence worldwide partly due to the population aging [1]. Although there are many treatments, such as external beam radiation therapy (EBRT) with androgen deprivation therapy (ADT) and low-dose-rate brachytherapy (BT) for localized PC, radical prostatectomy (RP) is still the most common option due to its good effect on tumor control [2]. However, patients who undergo RP may be faced with various complications, including urinary leakage, erectile dysfunction, bloody stools, nocturia, anxiety and depression, and general mental and physical function, among which postoperative urinary incontinence severely affects the quality of life and psychological endurance of patients [3–5]. Over time, urinary continence increases gradually. The majority of patients regain urinary continence at 12 months after RP, while only a minority of patients achieve satisfying immediate and early continence [6, 7]. Therefore, early recovery from postoperative urinary incontinence should be a matter of primary importance.

A series of preoperative, intraoperative and postoperative factors are related to the urinary continence after RP. It has been demonstrated that longer functional and membranous urinary length and preservation of neurovascular bundle contribute to the early urinary continence recovery [8–10]. However, with the development of surgery technology and robot-assisted laparoscopic radical prostatectomy, the influence of surgery itself on urinary continence has decreased [5]. However, some preoperative factors, such as obesity and comorbidities cannot be changed in a short time, and thus should not be the priority in postoperative urinary continence recovery [11]. Therefore, postoperative urinary continence recovery mainly depends on the postoperative treatments, including diet modification, bladder training, pelvic floor muscle exercises (PFME), biofeedback, and functional electrical stimulation [12, 13]. As previous study demonstrated, PFME conducted before RP may have a beneficial role on incontinence recovery rate at 3 months postoperatively [14].

It has been reported that the urinary continence of males is mainly related to the sphincteric competence, and pre- and postoperative bladder function [15]. Approximately two-thirds of postoperative urinary incontinence is related to urinary sphincter dysfunction and the other one-third is attributed to simultaneous dysfunction of the bladder and urinary sphincter. The urinary sphincter consists of the external and internal urinary sphincters, which are routinely removed during RP. Therefore, the status of the external urinary sphincter, which is often dysfunctional due to direct injury or nerve damage, is closely related to postoperative urinary continence restoration [16]. Restoration of external urinary sphincteric competence is a key step in urinary continence recovery after RP. As a noninvasive treatment, PFME is the first choice to improve urinary continence after RP with no side effects [17]. However, the significance of PFME in improving urinary continence after RP is still controversial. One of the common concerns is that the principles of PFME applied in men are based on those developed in women, which seems problematic due to the differences in anatomy [12]. In addition, there are currently no measurable indices for evaluating the effect of PFME.

Therefore, in the present study, we aimed to assess the significance of transrectal ultrasound_guided PFEM in improving urinary continence after RP and evaluate the role of postoperative external urinary sphincter status as a biomarker in predicting urinary continence.

## Materials and methods

### Participants and study design

In this retrospective study, 114 eligible patients with localized PC who underwent RP at Henan Cancer Hospital from November 2018 to April 2021 were analyzed. Patients who underwent laparoscopic or robotic-assisted radical prostatectomy were candidates, excluding those with preoperative urinary incontinence, lower urinary tract symptoms, neurogenic bladder, and a language barrier causing an inability to accurately express themselves and complete follow-up. All patients underwent the same surgical procedure during the operation. All patients were familiar with the study and signed the informed consent forms. All participants were followed up at 2 weeks and 1, 3, 6, and 12 months after RP. Urinary continence was evaluated by an oral questionnaire as previously described [18]. “No use of pads or no leakage of urine after catheter removal” was considered as urinary continence recovery. This study was approved by the Ethical Reviewing Committee of Henan Cancer Hospital.

The 114 eligible patients were divided into observation and control groups. Patients in the observation group and the control group started PFME before and after RP, respectively. Postoperative PFME was performed at Day 6 after RP, and the catheter was removed 2 weeks after RP. Before routine PFME, 64 patients in the control group were verbally guided by urologists, while the other 50 patients in the observation group received dual guidance by transrectal ultrasound and urologists. The demographic variables and other clinicopathological features are summarized in Table 1.

**Table 1:** Demographic and clinicopathological variables of patients in the observation and control group

Variables	Observation group (*N* = 50)	Control group (*N* = 64)	*P* value
Age (years)	67.5 ± 7.1	68.1 ± 5.7	0.665
BMI (kg/m^2^)	23.9 ± 2.4	24.2 ± 2.8	0.558
PSA (ng/ml)	4.2 ± 7.4	5.9 ± 7.8	0.243
Cholestenone (mmol/L)	4.5 ± 1.1	4.6 ± 0.8	0.641
Time of RP (min)	226.9 ± 91.2	243.6 ± 86.1	0.319
Intraoperative bleeding (ml)	186.2 ± 104.1	196.6 ± 159.7	0.693
Hospital stay (days)	16.8 ± 7.7	18.3 ± 6.6	0.274
Gleason scores			
≤ 7	24	29	0.775
≧8	26	35	
Neadjuvant therapy			0.413
No	10	17	
Yes	40	47	
TURP			
No	39	49	0.856
Yes	11	15	
Hypertension			
No	32	39	0.738
Yes	18	25	
Diabetes			
No	37	50	0.607
Yes	13	14	
Operation			0.433
LRP	31	35	
RARP	19	29	
Lymph node dissection			0.369
No	18	18	
Yes	32	46	

Measurement data were expressed using by mean ± SD, and analyzed using Student’s *t*-test. Categorical variables were detected by Chi-square test

*BMI* body mass index, *PSA* prostate specific antigen, *TURP* transurethral resection of the prostate, *LRP* laparoscopic radical prostatectomy, *RARP* robot-assisted radical prostatectomy

### Transrectal ultrasound and urologist_dually guided PFME

After signing the written consent, the candidates in the observation group were introduced to the program of transrectal ultrasound and urologist dually_guided PFME (TUUD-guided PFME). The procedures were as follows: first, transrectal ultrasound was used to locate the external urethral sphincter (EUS). When the patients performed PFME, the contraction of the EUS could be observed by the urologists and patients through the visualization system. Then, the urologists conducted digital rectal examination to feel the EUS contraction and guided the maximum EUS contraction induced by PFME. Finally, the patients were required to remember the state and keep training using the guided PFME. By doing so, the patients would achieve the correct PFME.

**Fig. 1:**
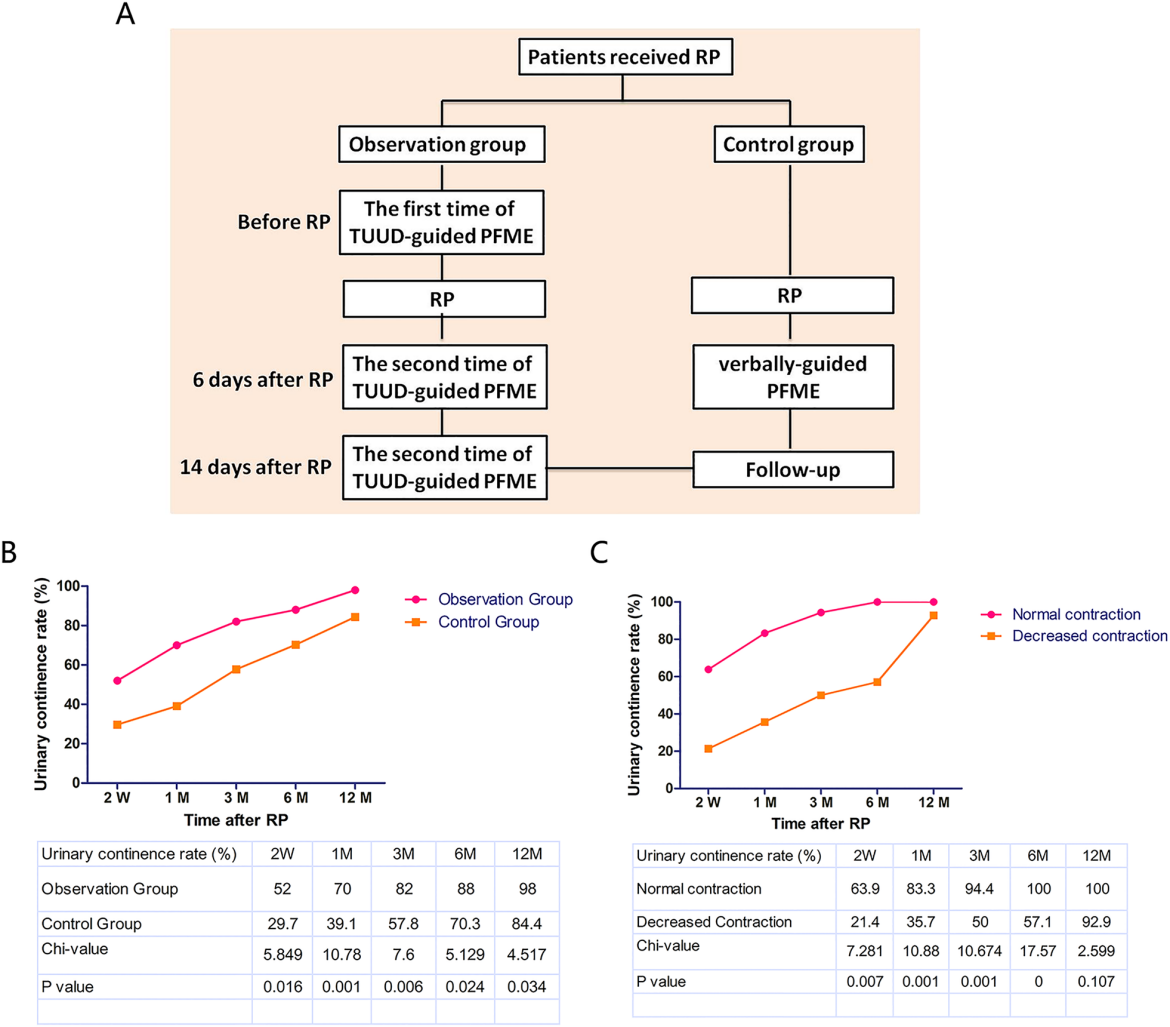
**A**: The flow diagram of TUUD-guided and verbally_guided PFME was exhibited. **B**: We evaluated the urinary continence rate at 5 time points, including 2 weeks and 1, 3, 6, and 12 months after RP, respectively. In both groups, the continence rate increased with time. **C**: 2-week and 1-, 3-, 6- and 12-month continence rate in the decreased group were obviously lower than those in the normal group (21.4% vs. 63.9%, 35.7% vs. 83.3, 50% vs. 94.4%, 57.1% vs. 100%, 92.9 vs. 100%)

The flow diagram of TUUD-guided and verbally_guided PFME is shown in Fig. 1A. Before RP, the patients in the observation group underwent the first time of TUUD-guided PFME session. Six days after RP, the second TUUD-guided PFME session was conducted, and the patients were required to start PFME training. The third TUUD-guided PFME session was conducted after catheter removal on Day 14. Patients in the control group began PFME on postoperative Day 6 with only verbal guidance.

### Evaluation of the contractile function of EUS

In the three transrectal ultrasound examinations, the contraction amplitude of the EUS was evaluated, and the methods of evaluation are described as follows: 1. the diameter of the urethra under the EUS was detected in a relaxed state and recorded as d1, d2 and d3. 2. The patients were instructed to perform PFME and then record the diameter of the urethra (d1’, d2’ and d3’) was recorded. 3. The difference between the two measurements (△1 = d1–d1’, △2 = d2–d2’, △3 = d3–d3’) was used to reflect the contraction amplitude of the EUS. The contractile function of the EUS was assessed by the ratio of △3 to △1 and defined as decreased if the ratio was less than 0.8 normal if the ratio was greater than 0.8.

### Statistical analysis

SPSS 19.0 (SPSS Inc., Chicago, IL, USA) and GraphPad (Prism 5.0) were used to conduct data analysis. Demographic and clinicopathological variables were analyzed by Student’s t and Chi-square tests. The relationship between urinary continence patient characteristics was analyzed using the Chi-square test. Logistic regression analyses were used to analyze the predictive factors for postoperative urinary continence. *P* < 0.05 was considered to be of significance.

## Results

In this study, we evaluated the urinary continence rate at 5 time points, including 2 weeks and 1, 3, 6, and 12 months after RP. In both groups, the continence rate increased with time (Fig. 1B). Statistical analysis showed that the 2-week and 1-, 3-, 6- and 12-month continence rates in the observation group were 52.0%, 70.0%, 82.0%, 88.0% and 98.0%, respectively, which were significantly higher than those in the control group (29.7%, 39.1%, 57.8%, 70.3% and 84.4%, respectively; all *p* < 0.05). Our data demonstrated that TUUD-guided PMME contributed to the immediate, early and long-term urinary continence after RP.

Next, we analyzed the relationship between the contractile function of the EUS and urinary continence in the observation group. As shown in Fig. 1C, the 2-week and 1-, 3-, 6- and 12-month continence rates in the decreased group were obviously lower than that in the normal group (21.4% vs. 63.9%, 35.7% vs. 83.3, 50% vs. 94.4%, 57.1% vs. 100%, 92.9 vs. 100%, respectively). Statistical analysis revealed a significant correlation between external urinary sphincter contraction and postoperative urinary continence at all time points except for 12 months.

Finally, we sought to determine the factors that affect the urinary continence after RP. Univariate logistic regression analysis revealed that transurethral resection of the prostate (TURP) and TUUD-guided PFME were associated factors for urinary continence at 2 weeks after RP (Table 2). Age, BMI and other variables had no significant relationship with 2-week urinary continence. Subsequently, we analyzed age, BMI, PSA, cholestenone, TURP and TUUD-guided PFME using a multivariate logistic regression model. Multivariate analysis demonstrated that TURP and TUUD-guided PFME were independent negative and positive independent factors for 2-week urinary continence, respectively (Table 2), as well as for 1-, 3-, 6- and 12-month urinary continence (Table 3). These data demonstrated that TUUD-guided PFME could be a positive prognostic factor for immediate, early and long-term urinary continence.

**Table 2:** Logistic regression analysis for urinary continence at 2 weeks after RP

Factors		Univariate analysis		Multivariate analysis	
		OR (95% CI)	*P* value	OR (95% CI)	*P* value
Age (year)		0.970 (0.914–1.030)	0.327	0.956 (0.894–1.021)	0.179
BMI (kg/m^2^)		0.986 (0.835–1.139)	0.844	0.984 (0.841–1.151)	0.840
PSA (ng/ml)		0.967 (0.915–1.022)	0.237	0.965 (0.906–1.027)	0.265
Cholestenone (mmol/L)		1.357 (0.907–2.031)	0.138	1.437 (0.934–2.211)	0.099
Time of RP (min)		0.999 (0.995–1.003)	0.659		
Intraoperative bleeding (ml)		1.000 (0.997–1.003)	0.906		
Hospital stay		0.950 (0.894–1.010)	0.101		
Gleason scores	≦7 vs ≧8	0.736 (0.346–1.564)	0.425	0.732 (0.324–1.652)	0.453
Neoadjuvant therapy	No vs yes	1.144 (0.469–2.789)	0.767		
TURP	No vs yes	0.377 (0.138–1.029)	0.057	0.293 (0.099–0.876)	0.027
Hypertension	No vs yes	0.858 (0.394–1.870)	0.700		
Diabetes	No vs yes	0.569 (0.225–1.441)	0.234		
Operation	LRP vs RARP	1.008 (0.471–2.156)	0.984		
Lymph node dissection	No vs yes	1.036 (0.461–2.328)	0.931		
Guidance	No vs yes	2.566 (1.186–5.550)	0.017	2.538 (1.124–5.732)	0.025

**Table 3:** Multiple logistic regression for urinary continence at 1, 3, 6 and 12 months after RP

Factors		Univariate analysis		Multivariate analysis	
		OR (95% CI)	*P* value	OR (95% CI)	*P* value
At 1 month					
TURP	No vs yes	0.384 (0.154–0.956)	0.04	0.301 (0.110–0.823)	0.019
Guidance	No vs yes	3.640 (1.658–7.989)	0.001	3.872 (1.681–8.920)	0.001
At 3 months					
TURP	No vs yes	0.286 (0.115–0.710)	0.007	0.212 (0.075–0.596)	0.003
Guidance	No vs yes	3.324 (1.385–7.979)	0.007	3.560 (1.364–9.290)	0.009
At 6 months					
TURP	No vs yes	0.329 (0.125–0.864)	0.024	0.283 (0.099–0.807)	0.018
Guidance	No vs yes	3.096 (1.131–8.484)	0.028	3.406 (1.158–10.019)	0.026
At 12 months					
TURP	No vs yes	0.129 (0.034–0.487)	0.002	0.087 (0.015–0.496)	0.006
Guidance	No vs yes	9.074 (1.120–73.487)	0.039	17.186 (1.580–186.95)	0.020

## Discussion

Urinary incontinence after RP is a common complication that has a severely adverse impact on patients’ quality of life and requires more care [19]. Scholars are constantly trying to find a reliable way to reduce the occurrence of urinary incontinence after RP. A series of strategies have been adopted to treat postoperative urinary incontinence, including PFME and surgical interventions [20]. Numerous studies have demonstrated that PFME is the most common conservative intervention for postoperative urinary incontinence [13, 21–23]. Although it is generally believed that PFM contractions can be achieved after verbal or written instructions for PFME, up to 50% of patients fail to achieve effective contractions after basic instructions [24]. Therefore, there is an urgent need to improve the effectiveness of PFME and to standardize guidance.

In the current study, based on research and practice, patients in the TUUD-guided PFME group achieved better urinary continence than those in the control group. Of the 50 patients in the observation group, 26 patients (52.0%) achieved urinary continence after catheter removal (2 weeks after RP), which was significantly higher than that in the control group (29.7%). In addition, urinary continence rates at 1, 3, 6 and 12 months after RP in the observation group were all higher than those in the control group. Although previous studies reported a controversial influence of PFME on improving postoperative urinary continence [24], our data demonstrated an effective influence of TUUD-guided PFME on immediate, early and long-term urinary continence recovery after RP. To improve postoperative urinary continence, modified PFME was administered. Centemero, A et al. initiated PFME before RP and observed an improved early continence rate compared with postoperative PFME [25]. Seong J. et al. developed a series of nomograms to predict the recovery of urinary continence after RP and achieved significant improvement in continence [26]. Ultrasound-guided PFME was reported to not only improve the early incontinence recovery with a continence rate of 52.8% within 30 days after RP, but also the prolonged urinary incontinence occurring > 1 year later [27, 28]. Compared with these studies, our study revealed outstanding immediate and early continence rates under postoperative TUUD-guided PFME, as well as late continence rates. By means of transrectal ultrasound, we clearly observed the EUS contraction and guided the maximum external urethral sphincter contraction training by the digital rectal examination during PFME, which indicated that correct PFME is vital for urinary continence recovery after RP. Furthermore, correct PFME would provide long-term benefits in urinary continence for patients. However, a major bias in timing of PFME training in the two groups in this retrospective study existed, and the role of TUUD-guided PFME in improving urinary continence recovery after RP still needed to be further verified in prospective study in which synchronous PFME was required.

At the time of transrectal ultrasound examination, the maximum EUS contraction was measured. According to the algorithms we formulated, the contractile function of EUS after PFME was classified into decreased and normal. In the transrectal ultrasound and urologist_dually guided PFME cohort, the 2-week, 1-month, 3-month, 6-month and 12-month continence rates in the decreased group were obviously lower than those in the normal group, which indicated that the EUS contraction amplitude was a promising index for predicting postoperative urinary continence after RP. Stéphanie J. et al. reported that the thickness, cross-sectional area and volume of the urethral sphincter were increased in women after a 12-week group PFM rehabilitation intervention [29], which was similar to our study. It has been reported that > 90% of patients with long-term urinary incontinence have EUS impairment [30]. Therefore, a decreased contraction after precise PFME reflected the possibility of EUS impairment that may need other treatments. With the help of transrectal ultrasound, detecting EUS contraction was easy. Meanwhile, compared with determination of the membranous urethral length determined by MRI, the EUS-guided contraction by ultrasound required a lower cost. In addition, with the application of micro-ultrasound in urology, assessment of EUS contraction would be more precise and easier in the future [31]. However, because of the limited sample size of this study, this assessment method of the EUS contraction requires a large sample size for further verification.

Finally, we conducted an analysis of factors influencing immediate, early and long-term urinary continence after RP. Our data revealed that TURP and TUUD-guided PFME were associated factors for urinary continence at 2 weeks and 1, 3, 6 and 12 months after RP. TURP has been verified by previous studies to have adverse influences on urinary continence possibly because of the fibrosis of periurethral tissues that may inhibit EUS function [32]. Therefore, maintaining the integrity of the EUS and strengthening EUS training are vital during the perioperative period. TUUD-guided PFME was verified to be an independent factor in predicting immediate, early and long-term continence, which was partly because of the quick and correct recovery of the EUS function under the current model of PFME.

In conclusion, our data verified the effectiveness of TUUD-guided PFME in improving urinary continence after RP and the possibility of using EUS contraction in predicting urinary continence. In addition, no assistive devices were used in the current model, and training without equipment was carried out before the urinary catheter was not removed, which improved the rate of voiding control after removal of the urinary catheter.

There were still some shortcomings in this study. First, this was only a retrospective single-center study with a small sample size. Multicenter, large-scale and prospective research is needed to further verify the effectiveness of current model of PFME. Second, this study only focused on the recovery of the patient’s body, and did not introduce psychological status evaluation, which needs to be assessed in the future.

## Conclusions

Transrectal ultrasound and urologist-dually guided PFME had significant role in improving immediate, early and long-term urinary continence after RP, and acted as an independent prognostic factor.

## Data Availability

The data that support the findings of this study are available from the corresponding author upon reasonable request.

## Abbreviations

ADT: Androgen deprivation therapy
BT: Low-dose-rate brachytherapy
EBRT: External beam radiation therapy
EUS: External urethral sphincter
PC: Prostate cancer
PFME: Pelvic floor muscle exercises
RP: Radical prostatectomy
TUUD-guided PFME: Urologist-dually guided PFME
