# Correction: The significance of transrectal ultrasound and urologist_dually guided pelvic floor muscle exercise in improving urinary continence after radical prostatectomy

**DOI:** 10.1186/s40001-023-01163-x

**Published:** 2023-06-24

**Authors:** Yin Huaqi, Du Zheng, Ma Yongkang, Zhao Shiming, Sun Zhenghui, Wang Zhiwei, Li Congyu, Li Qian, Dong Bingqi, Zhu Mingkai, Zhu Chaoshuai, Peng Jiangshan, Yang Tiejun

**Affiliations:** 1 Department of Urology, The Affiliated Cancer Hospital of Zhengzhou University, Henan Cancer Hospital, No.127, Dong Ming Road, Zhengzhou, 450000 China; 2 Department of Ultrasound, The Affiliated Cancer Hospital of Zhengzhou University, Henan Cancer Hospital, No.127, Dong Ming Road, Zhengzhou, 450000 China

**Correction: European Journal of Medical Research (2023) 28:171** **https://doi.org/10.1186/s40001-023-01133-3**

In the original publication of the article [1], the affiliation details of the authors were incorrect. The corrected affiliations were given in this Correction article. The original article has been corrected.

## References

[1] Huaqi Y, Zheng D, Yongkang M, Shiming Z, Zhenghui S, Zhiwei W, Congyu L, Qian L, Bingqi D, Mingkai Z, Chaoshuai Z, Jiangshan P, Tiejun Y (2023). The significance of transrectal ultrasound and urologist_dually guided pelvic floor muscle exercise in improving urinary continence after radical prostatectomy. Eur J Med Res.

